# CELEBRATING 50 YEARS OF AGING RESEARCH: A CHAT WITH NIA SENIOR LEADERSHIP

**DOI:** 10.1093/geroni/igae098.0329

**Published:** 2024-12-31

**Authors:** Amy Kelley, Amy Kelley

**Affiliations:** Chair; Discussant

## Abstract

The National Institute on Aging (NIA) at the National Institutes of Health, Department of Health and Human Services, is the federally designated lead agency on aging research and supports research to understand the nature of aging and to extend the healthy, active years of life. 2024 marks NIA’s 50th anniversary, a milestone which prompts reflection on both past and present discoveries resulting from NIA-funded research. In the decades since its establishment, NIA has propelled advances across multiple domains of aging research, including basic, clinical, neuroscience, and behavioral and social research. Looking to the future, NIA seeks to drive further advances in these areas – with input on strategic priorities from investigators, advocates, and the public – to enable healthier aging for all. This symposium will provide a forum for exploration of NIA-funded research advances, as well as current and future research priorities of NIA’s scientific divisions, and review of the NIA budget and its implications for the research community. A question-and-answer session with NIA senior staff will follow these remarks.

